# A pragmatic, randomised, open-label, multi-centre UK registry-facilitated trial evaluating the effect of high-dose cholecalciferol on mortality in patients on dialysis: the SIMPLIFIED trial

**DOI:** 10.1186/s13063-025-09134-9

**Published:** 2025-10-15

**Authors:** Toby J. L. Humphrey, Fergus J. Caskey, David C. Wheeler, Ian B. Wilkinson, Edward C. F. Wilson, Adam P. Wagner, Francis Dowling, Barbara Vencilas, Molly Cui, Simon J. Bond, Thomas F. Hiemstra, Rona M. Smith

**Affiliations:** 1 Cambridge University Hospitals NHS Foundation Trust, Cambridge, UK; 2 Bristol Medical School, University of Bristol, Bristol, UK; 3 Centre for Kidney and Bladder Research, University College London, London, UK; 4 University of Cambridge, Cambridge, UK; 5 Exeter Medical School, University of Exeter, Exeter, UK; 6 Norwich Medical School, University of East Anglia, Norwich, UK; 7 National Institute for Health and Care Research (NIHR) Applied Research Collaboration East of England (NIHR ARC EoE), Cambridge, UK

**Keywords:** Dialysis, Cholecalciferol, Mortality, Clinical trial, End stage kidney disease (ESKD)

## Abstract

**Background:**

Vitamin D deficiency is common in patients with end stage kidney disease (ESKD) and is a strong predictor of death from cardiovascular disease, infections and cancer. Currently only 68% of patients in the UK survive 3 years or more on dialysis. Vitamin D replacement typically utilises activated (1α-hydroxylated) vitamin D compounds allowing for impaired renal activation. However, this approach increases blood calcium concentrations and may paradoxically accelerate vitamin D catabolism. Contemporary treatment guidelines recommend replacement with inactive (unhydroxylated) vitamin D_3_ (cholecalciferol), instead relying on autocrine and paracrine vitamin D activation in target tissues. However, the impact of this approach on clinical outcomes has not yet been tested in adequately powered trials.

**Methods:**

The SIMPLIFIED (Survival Improvement with Cholecalciferol in Patients on Dialysis) trial will test whether supplementation with high-dose cholecalciferol improves survival. Adult dialysis patients will be recruited from one of 72 UK renal centres, by local clinicians and research nurses, and randomly assigned in a 1:1 ratio to high-dose open-label cholecalciferol or standard care, stratified by site. Trial outcomes will be captured using existing data sources including Office of National Statistics (ONS) for deaths and Hospital Episode Statistics (HES) for secondary endpoints. The primary endpoint of the trial is all-cause mortality. Key secondary endpoints include survival free from cardiovascular events, infections and cancers, and quality of life and cost-effectiveness measures utilising the Eq5D tool. Severe adverse event line listings will be generated from HES data for safety reporting. In this superiority trial, it is anticipated that approximately 4200 patients in total will need to be enrolled to observe 2200 deaths over a period of about 7 years, which will yield 90% power to demonstrate a hazard ratio of 0.87.

**Discussion:**

Outcomes for patients on dialysis remain very poor. The pragmatic design of this study leveraging routinely collected data streamlines trial conduct, reducing burden on patients and local site study staff. Furthermore, since cholecalciferol is a low-cost and easy to administer intervention, evidence of benefit could be readily incorporated into dialysis care across healthcare systems globally.

**Trial registration:**

EudraCT Number: 2015-005003-88; ISRCTN Number: 15087616 (registration date 30th December 2015) (https://doi.org/10.1186/ISRCTN15087616). Recruitment commenced in March 2017.

## Administrative information

Note: the numbers in curly brackets in this protocol refer to SPIRIT checklist item numbers. The order of the items has been modified to group similar items (see http://www.equator-network.org/reporting-guidelines/spirit-2013-statement-defining-standard-protocol-items-for-clinical-trials/).

**Table:** 

Title {1}	Survival Improvement with Cholecalciferol in Patients on Dialysis: The SIMPLIFIED registry trial
Trial registration {2a and 2b}.	EudraCT Number: 2015-005003-88 ISRCTN Number: 15087616
Protocol version {3}	Protocol V11.0 6^th^ February 2025
Funding {4}	National Institute of Health Research (NIHR). Award ID: 14/49/127
Author details {5a}	1. Cambridge University Hospitals NHS Foundation Trust, Cambridge, UK 2. Bristol Medical School, University of Bristol, Bristol, UK 3. Centre for Kidney and Bladder Research, University College London, UK 4. University of Cambridge, Cambridge, UK 5. Exeter Medical School, University of Exeter, UK 6. Norwich Medical School, University of East Anglia, UK 7. National Institute for Health and Care Research (NIHR) Applied Research Collaboration East of England (NIHR ARC EoE), UK
Name and contact information for the trial sponsor {5b}	Mr Stephen Kelleher Cambridge University Hospitals NHS Foundation Trust Email: cuh.research@nhs.net
Role of sponsor {5c}	The trial is sponsored by Cambridge University Hospitals NHS Foundation Trust and University of Cambridge. This is an academic initiated study, and decisions regarding the design and conduct of the study rest with the study team and sponsor. The funder will be notified of results ahead of publication.

## Introduction

### Background and rationale {6a}

In the United Kingdom (UK), nearly 30,000 people receive regular dialysis, and 6000 new patients start dialysis each year. Although this is a life-saving treatment, dialysis-requiring kidney failure carries a worse prognosis than most malignancies [1], with a mean 3-year survival of only 68%. The mean age of UK dialysis recipients is 65 years, and after 4 years on dialysis, fewer than 40% of those aged 65 and over survive. Those aged 60–69 years have nine times the relative risk of death compared to the UK general population. The three leading causes of death are cardiovascular disease, infections and malignancy [1]. Patients receiving maintenance dialysis experience significant physical, emotional, mental and psychological impairments which are reflected in health-related quality of life (HRQoL) scores [2].

Treatment of patients with end stage kidney disease (ESKD) is disproportionately costly, accounting for up to 2% of the National Health Service (NHS) healthcare budget whilst representing only 0.05% of the population [3]. Despite this, nephrology has historically lagged behind most disciplines in testing interventions in randomised trials [4]. A key obstacle to trials in dialysis is the readiness with which treatment guidelines are accepted into clinical practice without adequate prior evaluation or introduction in a randomised manner. A wide range of interventions currently employed in this population in the NHS, at considerable cost, are aimed at improving survival and quality of life, but have either failed to show any benefit (erythropoiesis-stimulating agents [5], lipid-lowering agents [6], calcimimetics [7]) or have not been adequately evaluated in randomised trials (phosphate-lowering agents or active vitamin D compounds).

In patients with kidney disease, plasma 25(OH)D, the main circulating form of vitamin D, begins to decline when the glomerular filtration rate falls below 45 ml/min/1.73 m^2^. Once patients reach dialysis, only a small minority are vitamin D sufficient. In a cohort of 6518 German dialysis patients, 76% were vitamin D deficient [8]. Despite the introduction of national and international guidelines recommending the measurement and supplementation of vitamin D in patients on dialysis, reports over the past decade consistently show vitamin D deficiency in this population [9–11]. Dialysis patients are typically treated with active 1α-hydroxylated vitamin D compounds, such as alfacalcidol or paricalcitol, a practice inherited from the now disproven belief that activation through 1α-hydroxylation occurred only in the kidneys. It is now known that synthetic and catabolic hydroxylases are ubiquitously expressed and the vitamin D endocrine system is involved in a wide range of cellular functions including cellular growth, proliferation, apoptosis and inflammation [12]. Whilst the use of 1α-hydroxylated compounds is effective in lowering parathyroid hormone levels, their use is associated with hypercalcaemia, worsening hyperphosphataemia, potential acceleration of vascular calcification and potent induction of vitamin D catabolism, thus potentially worsening systemic and cellular vitamin D deficiency. In contrast, the administration of cholecalciferol would circumvent all of these limitations and is both low-cost and readily available.

A large body of epidemiological evidence supports a role for vitamin D in reducing cardiovascular disease, malignancy and infections. In otherwise healthy persons, deficiency is strongly associated with cardiovascular, cancer and all-cause mortality, and key pathways in host defence are dependent upon vitamin D sufficiency [13]; each 25 nmol/L reduction in 25(OH)D was associated with a 16% increase in all-cause mortality in a large meta-analysis including data from 849,412 subjects. The pooled relative risk (RR) was 1.35 (95% CI 1.13 to 1.61) for death from cardiovascular disease, 1.14 (1.01 to 1.29) for death from cancer, 1.30 (1.07 to 1.59) for non-vascular, non-cancer death and 1.35 (1.22 to 1.49) for all-cause mortality [14].

However, two large trials with over 30,000 participants have not shown significant benefit of vitamin D supplementation in patients without chronic kidney disease (CKD) [15, 16]. The VIDA (Vitamin D Assessment) study did not demonstrate any significant reduction in incident cardiovascular disease or death in those treated with cholecalciferol versus placebo (adjusted hazard ratio, 1.02; 95% CI, 0.87–1.20) [15]. The VITAL study demonstrated that vitamin D did not lower the incidence of major cardiovascular events (HR 0.97; 95% CI, 0.85–1.12) or cancer incidence (HR 0.96; 95% CI, 0.88–1.06) [17]. However in patients with kidney failure, one small trial of 126 patients randomised to high-dose ergocalciferol or placebo demonstrated reduced cardiovascular events in the ergocalciferol arm (HR 0.37, 95% CI 0.14–1.0) [17].

The Kidney Disease Improving Global Outcomes (KDIGO) initiative accept that current evidence is lacking and identifies vitamin D supplementation in dialysis as a key research objective, but nevertheless argues for its use on the basis that the intervention is safe and inexpensive [18]. Caution is necessary, however, when extrapolating from epidemiological data. Observational data similarly supported the use of antioxidant vitamins including vitamins C and E, which were found to be of no benefit or even harmful in adequately powered interventional trials [19, 20]. Widespread supplementation with cholecalciferol should therefore be rigorously tested in an adequately powered randomised trial prior to implementation.

At conception of this trial, a survey of UK renal units illustrated that vitamin D was neither routinely measured nor supplemented in the UK dialysis population. In contrast to other countries where supplementation is already more prevalent, this lag in implementing existing guidelines provides the opportunity to test the hypothesis that cholecalciferol improves survival and quality of life in a population that is mostly treatment naïve. The SIMPLIFIED trial will address this clear unmet need for an adequately powered clinical trial to assess the impact of cholecalciferol on quality of life and survival in ESKD before evidence–poor guideline-driven vitamin D replacement becomes entrenched in clinical practice.

### Objectives {7}

#### Primary objective

To determine the effect of cholecalciferol (60,000 IU fortnightly) on patient survival.

#### Secondary objectives

To determine the effect of cholecalciferol (60,000 IU fortnightly) on hospital admissions due to cardiovascular events, infections or fracture, cancer incidence, quality of life and evaluate the cost-effectiveness of cholecalciferol from the perspective of the NHS.

### Trial design {8}

SIMPLIFIED is a prospective, randomised, open-label, pragmatic, multi-centre registry-facilitated superiority trial of cholecalciferol (also known as colecalciferol) versus standard care in patients receiving dialysis.

## Methods: participants, interventions and outcomes

### Study setting {9}

There are 72 renal units across the UK that contribute data to the UK Renal Registry (UKRR). It is anticipated that the SIMPLIFIED trial will be conducted in up to 65 of these units.

### Eligibility criteria {10}

#### Inclusion criteria

To be included in the trial the patient must:

- Have given written informed consent to participate
- Be aged 18 years or over
- A UK resident
- Has ESKD requiring dialysis

#### Exclusion criteria

The presence of any of the following will preclude patient inclusion:

- Treatment with high-dose (> 1000 IU/day) cholecalciferol or ergocalciferol in the last 30 days
- Persistent hypercalcaemia (corrected calcium > 2.62 mmol/l on three separate and sequential occasions without precipitating cause)
- Hypersensitivity to cholecalciferol or any of the excipients of the product
- Life expectancy < 6 months
- Women who are pregnant/planning to become pregnant or are breastfeeding
- Opted out from contributing data to the UK Renal Registry (UKRR)

### Who will take informed consent? {26a}

Consent will be obtained by the principal investigator (PI) or by a suitably qualified and delegated health professional and member of the research team at each site. The patient will be provided with the approved Patient Information Sheet at a routine dialysis appointment. If the patient is interested in the study, a member of the study team will discuss the study with them and answer any questions they may have, usually at a subsequent dialysis appointment but trial discussions could also take place by telephone. The investigator or designee will obtain written, informed consent from each patient before any study procedures are undertaken.

In specific cases, such as when access to dialysis units is restricted or patients receive home dialysis, consent can be taken remotely via telephone or other audio/visual teleconference methods by the PI or a delegated team member. If remote consent is obtained, then the copy of the Informed Consent Form sent to the patient via post is signed by the participant and then sent back for signature by the PI or delegated team member who took the consent remotely as soon as it is received. Face to face consent will still be the primary method of consent and this remote method should only be used when face to face is not possible.

### Additional consent provisions for collection and use of participant data and biological specimens {26b}

Trial participants will provide explicit consent for the collection and use of patient identifiable data (name, date of birth, gender, NHS number or equivalent, home address and postcode, telephone number and/or email address) for the purposes of the conduct of the trial. Patient identifiable data will be stored separately from anonymised trial data on a secure server hosted within University of Cambridge. This data is required for linkage with routinely collected datasets obtained via NHS England and devolved nation equivalents to gather data on primary and secondary endpoints, deaths and hospital admissions respectively, and is therefore imperative for the conduct of the trial.

## Interventions

### Explanation for the choice of comparators {6b}

Participants randomised to the control group will receive standard care based on local treatment guidelines but will not be permitted to receive cholecalciferol or ergocalciferol > 1000 IU per day for the duration of the study, unless vitamin D deficiency-related osteomalacia is diagnosed.

### Intervention description {11a}

Patients randomised to receive cholecalciferol will be prescribed cholecalciferol 60,000 IU fortnightly, in addition to standard care. Where their standard care already involves the administration of low-dose cholecalciferol or ergocalciferol (≤ 1000 IU/day), the relevant prescription must be discontinued upon randomisation to the cholecalciferol group and prior to the administration of the first trial dose.

### Criteria for discontinuing or modifying allocated interventions {11b}

**Fig. 1:**
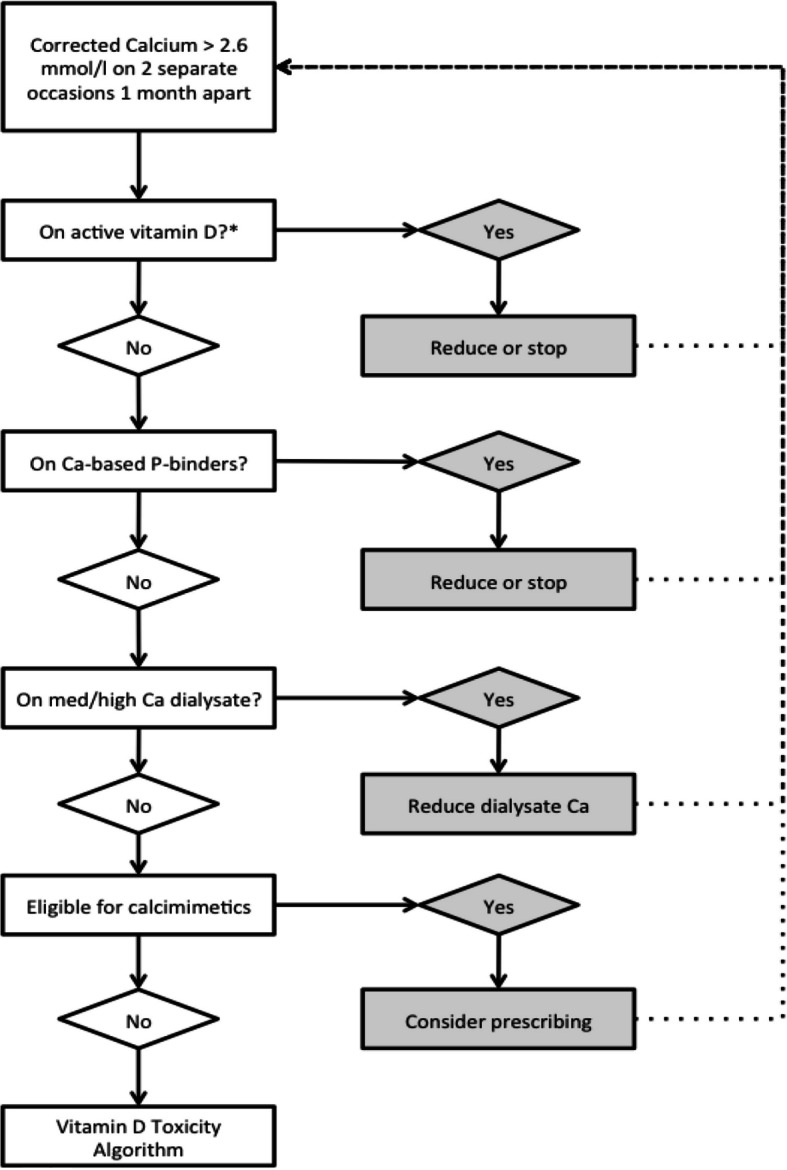
Hypercalcaemia algorithm. Ca, calcium; P, phosphate. Given that cholecalciferol treatment should at least partially restore endogenous calcitriol synthesis, the reduction or withdrawal of active vitamin D compounds should be prioritised. *Active vitamin D: all 1α-hydroxylated vitamin D compounds including (but not limited to) alfacalcidol, paricalcitol, calcitriol and 22-oxacalcitriol

**Fig. 2:**
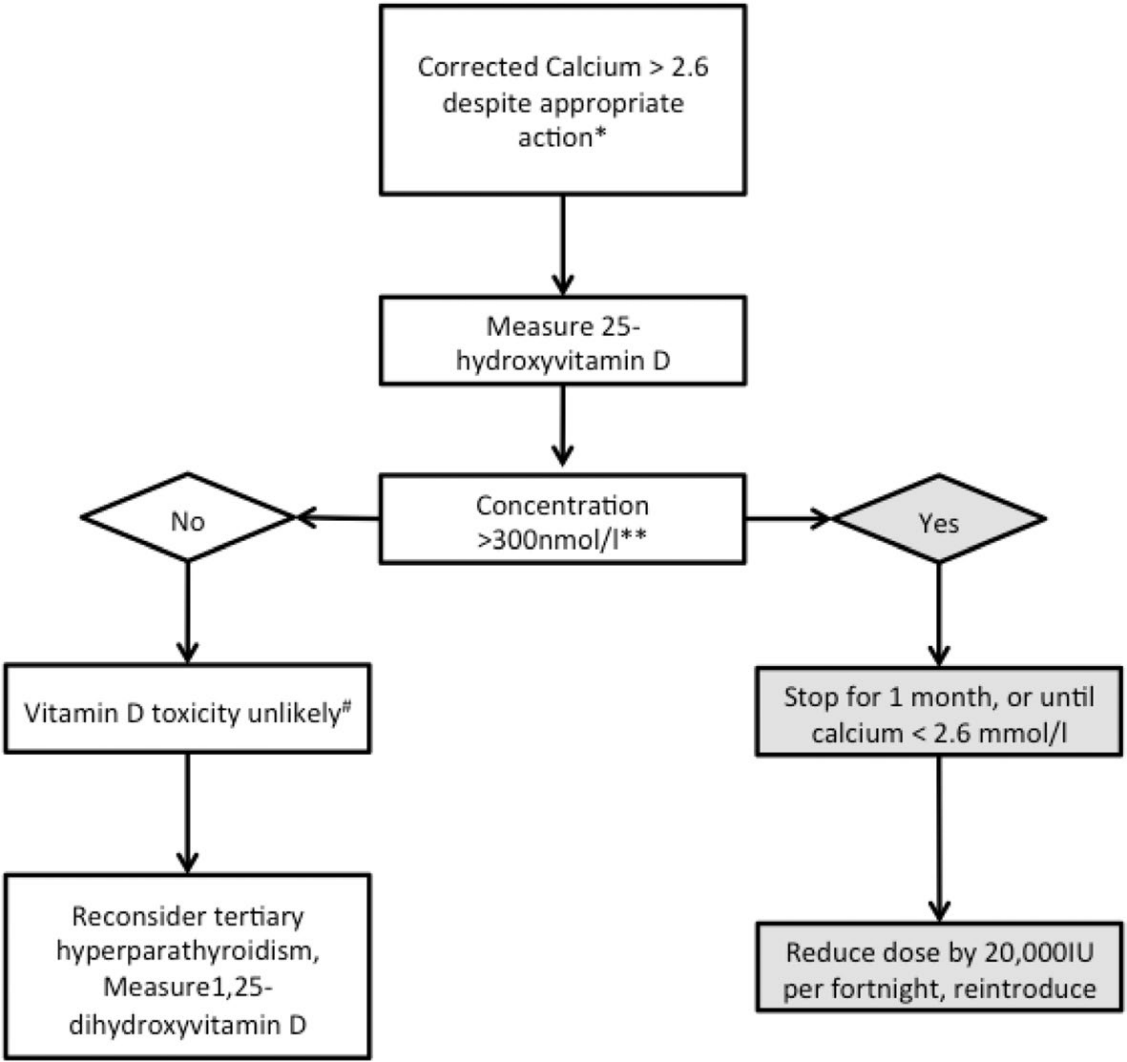
Suspected vitamin D toxicity. If all identifiable causes of hypercalcaemia (Fig. 1) have been excluded or addressed, vitamin D toxicity should be considered in patients receiving cholecalciferol. This includes discontinuation of 1α-hydroxylated vitamin D compounds, since toxicity results from increased circulating calcitriol concentrations. Therefore, vitamin D toxicity cannot be attributed to cholecalciferol in the presence of concomitant treatment with 1α-hydroxylated compounds

Circumstances may arise under which cholecalciferol will be temporarily discontinued or dose reduced for participants in the interventional arm. Discontinuation of trial drug does not constitute withdrawal from the trial. Discontinuation may occur under the following circumstances:

- Vitamin D toxicity. Hypercalcaemia (Fig. 1) and vitamin D toxicity (Fig. 2) algorithms are illustrated below, and cholecalciferol may be discontinued if there is persistent toxicity despite prior institution of the dose change recommendations.
- Severe adverse reactions (SAR or SUSAR) or adverse reactions which in the opinion of the PI requires discontinuation of cholecalciferol.

Participants may withdraw consent for continuation in the trial at any point. Withdrawal will constitute discontinuation of the trial drug for those in the cholecalciferol arm. In the event that a participant withdraws from the trial, ongoing data capture through linkage will continue unless the participant also withdraws from data linkage and any future data capture.

Trial participants who receive a kidney transplant or become pregnant should be withdrawn from cholecalciferol if in that group; however, they will remain within the trial providing ongoing data captured through linkage unless the participant withdraws from data linkage.

### Strategies to improve adherence to interventions {11c}

SIMPLIFIED has been designed as a pragmatic trial, and as such cholecalciferol will not require trial-specific dispensing, labelling or accountability records. However, it is anticipated that the majority of patients enrolled in the trial will be receiving in-centre haemodialysis, and cholecalciferol will be administered as part of the dialysis prescription where possible, and hence will be supervised.

Plasma vitamin D levels will also be measured during the first year of the trial. Samples will be taken 4 months (collected in a window of −1 week and + 5 weeks) after the baseline visit for the first 230 patients enrolled into the trial to ensure that there is separation in levels between the cholecalciferol and control groups.

### Relevant concomitant care permitted or prohibited during the trial {11d}

With the exception of cholecalciferol or ergocalciferol, there is no contra-indicated concomitant therapy. Patients randomised to the cholecalciferol group, whose standard care already involves the administration of low-dose cholecalciferol or ergocalciferol (≤ 1000 IU/day), must discontinue these medications upon randomisation to the cholecalciferol group and prior to the administration of the first trial dose.

All patients will be permitted to continue treatment with any active (1α-hydroxylated) compounds, including alfacalcidol, calcitriol, paricalcitol and 22-oxacalcitriol. It is anticipated that cholecalciferol 60,000 IU fortnightly will reduce or remove the requirement for active vitamin D. Therefore, prescriptions for active vitamin D preparations should be assessed regularly along with routine dialysis reviews as per local policy whilst patients are enrolled in the trial.

### Provisions for post-trial care {30}

Should this intervention improve patient survival, cholecalciferol is a low-cost, easy to administer therapy, and it is anticipated that it could be rapidly rolled out to the UK dialysis population. For participants, whilst in the trial, Cambridge University Hospitals NHS Foundation Trust, as a member of the NHS Clinical Negligence Scheme for Trusts, will accept full financial liability for harm caused to participants in the clinical trial caused through the negligence of its employees and honorary contract holders. There are no specific arrangements for compensation should a participant be harmed through participation in the trial, if no-one has acted negligently. The University of Cambridge will arrange insurance for negligent harm caused as a result of protocol design and for non-negligent harm arising through participation in the clinical trial.

### Outcomes {12}

#### Primary outcome measure

The primary outcome is all-cause mortality. Deaths will be captured from Office National Statistics (ONS) datasets.

#### Secondary outcome measures

- Health-related quality of life assessed by EQ-5D-5L
- Composite cardiovascular events defined as cardiovascular death, acute coronary syndrome (ACS), heart failure, cardiac arrhythmia admissions and stroke requiring hospital admission from Hospital Episode Statistics (HES) data*
- Infections requiring hospital admission from HES data*
- Cancer incidence
- Fractures requiring hospital admission from HES data*
- Cost-effectiveness of cholecalciferol from the perspective of the NHS

*The statistical analysis plan will contain lists of specific HES codes which constitute each of the secondary endpoints.

### Participant timeline {13}

**Fig. 3:**
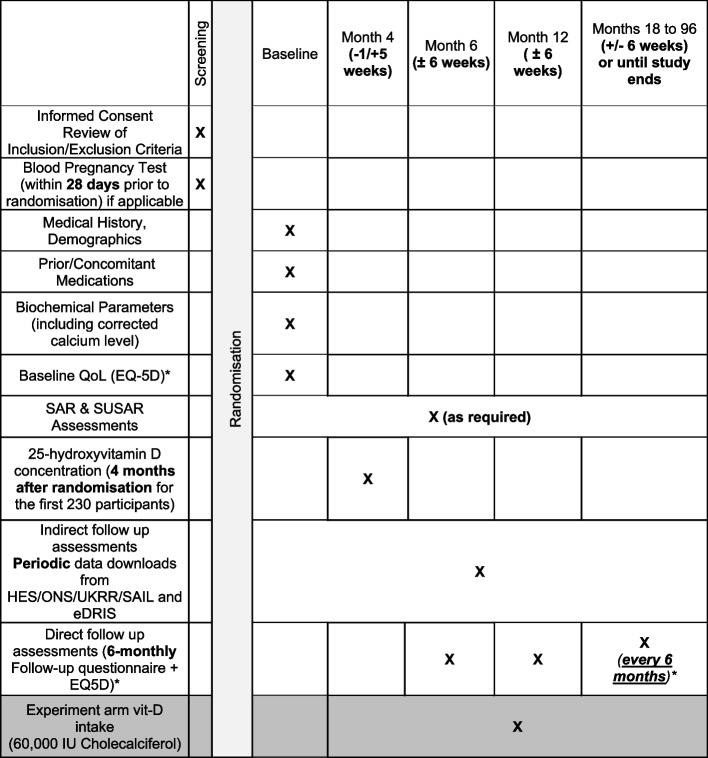
Assessment schedule. *Eq5D data collection ceased in April 2023. QoL, quality of life; SAR, serious adverse reaction; SUSAR, suspected unexpected serious adverse event; HES, Hospital Episode Statistics; ONS, Office of National Statistics; UKKR, UK Renal Registry; SAIL, Secure Anonymised Information Linkage; eDRIS, Electronic Data Research and Innovation Service

SIMPLIFIED is a pragmatic trial designed to exploit existing data sources to maximise efficiency and minimise research cost. As such no dedicated study visits outside of routine care are planned (Fig. 3). Many variables often captured face to face or during trial visits will be captured indirectly using a variety of routinely collected data sources. Data on the primary endpoint (death) will be captured via regular data tranches from Office of National Statistics (ONS). Data on secondary endpoints will be captured using Hospital Episode Statistics (HES) data downloads (or equivalent in Scotland and Wales), obtained regularly for the trial population. Laboratory data will be obtained from the results of routinely collected blood tests via the UK Renal Registry (UKRR) on an on-going basis. Calcium and phosphate concentrations are measured regularly in all UK dialysis patients as part of standard care, and these data are already submitted to the UKRR. Monitoring for hypercalcaemia will be driven by routine clinical practice and management based on local hypercalcaemia guidelines and practices.

### Sample size {14}

The SIMPLIFIED study is an event-driven trial. It is estimated that the control arm has a 3-year survival of 68% [1]. It is estimated that randomisation of 4200 patients would yield 2200 primary outcome events (deaths) over 7–8.5 years, resulting in 90% power at 5% significance (two-sided) assuming a hazard ratio of 0.87. Dropout is assumed to be negligible given the national death registry data source for primary endpoint collection.

### Recruitment {15}

SIMPLIFIED is a pragmatic, registry-facilitated study with broad inclusion criteria and limited exclusion criteria to facilitate recruitment of 4200 patients requiring dialysis and ensure that results are applicable to the general dialysis population. There are 72 renal units in the UK and it is anticipated that SIMPLIFIED will recruit from up to 65 of these. In September 2021, ethical approval was granted to enable patients to be approached and consented remotely. This measure was introduced in response to the COVID-19 pandemic which restricted movement of research nurses to satellite dialysis units, and also to encourage patients who receive dialysis outside of a unit, such as those receiving home haemodialysis or peritoneal dialysis, who are underrepresented in dialysis trials to enrol.

## Assignment of interventions: allocation

### Sequence generation {16a}

Patients will be randomised to the cholecalciferol treatment group or control group in a 1:1 ratio using a central computerised randomisation system.

### Concealment mechanism {16b}

This is an open-label study. Immediate allocation of treatment will be performed, with documentation of the decision in a confirmatory email automatically generated by the randomisation system.

### Implementation {16c}

Eligible participants will be randomised by local investigators using the web-based randomisation system embedded within the bespoke trial database, hosted by University of Cambridge. Blocked randomisation using a random block size of either 2 or 4, stratified by site, will be used. The randomisation is open-label, so the treatment assigned will be given explicitly and not blinded in any way.

## Assignment of interventions: blinding

### Who will be blinded {17a}

This is an open-label trial with patients and investigators aware of treatment allocation at an individual patient level. However, aggregate statistics broken down by treatment arms are only available to the IDMC.

### Procedure for unblinding if needed {17b}

Unblinding is not required as this is an open-label study.

## Data collection and management

### Plans for assessment and collection of outcomes {18a}

SIMPLIFIED is a pragmatic trial designed to exploit existing data sources to maximise efficiency and minimise research cost. Aside from a single baseline visit, when data is entered into an eCRF, no dedicated study visits outside of routine care are planned.

In the SIMPLIFIED trial, many variables often captured face to face or during trial visits will be captured indirectly using a variety of data sources including Office for National Statistics (ONS) for the primary endpoint death, Hospital Episode Statistics (HES) or equivalents in Scotland and Wales for secondary endpoints including cardiovascular events, severe infections and fractures, and the UK Renal Registry (UKRR) for biochemical data, such as calcium levels, which are collected and submitted to the UKRR as part of routine care. In order to evaluate the impact of the intervention on quality of life, 6-monthly EQ-5D questionnaires will be completed by participants either via a telephone call with the central study team in Cambridge, using a smartphone/web portal interface, or on paper via the post. Trial participants will provide explicit consent to the use of patient identifiable data for the purposes of the conduct of the trial. Patient identifiable data will be stored separately from anonymised trial data on a secure server hosted within University of Cambridge.

### Plans to promote participant retention and complete follow-up {18b}

SIMPLIFIED was designed to place minimal burden on participants, recognising the significant impact that dialysis already has on patients’ life, with the intention of promoting retention throughout the trial. However, it is important to collect the impact of cholecalciferol on quality of life, and therefore patients were offered the option of receiving 6-monthly EQ-5D questionnaires through the post, a smartphone/web EQ-5D data entry portal or to complete them via a telephone call with support from central study staff. For those patients who withdraw from their randomised treatment allocation in the trial, they are asked to consent to ongoing long-term follow-up via collection of routinely collected data which does not place any burden on them but provides valuable information on the long-term impact of the intervention.

### Data management {19}

Baseline data will be entered at site into an eCRF. Logical checks have been incorporated into each data field to minimise erroneous data entry. All trial data in the eCRF must be extracted from and be consistent with the relevant source documents. All subsequent data will be collected centrally. EQ-5D questionnaires will be delivered by central study staff either via telephone or post, and results entered directly into the study database, or by participants directly entering responses to EQ-5D questions via a smartphone/web interface linked directly with the study database. Data to capture all other endpoints including deaths and hospitalisations will require periodic uploads of the trial cohort to NHS England and equivalents in the devolved nations. Data received on deaths from ONS and hospitalisations from HES will be received into a secure data hosting area, processed and analysed in conjunction with study data collected via the more conventional methods. To identify patients in whom events have occurred, ICD-10 codes within HES will be flagged to identify subjects who have experienced an event (such as myocardial infarction, heart failure, stroke). A detailed description of relevant ICD-10 codes and classification will be included as an appendix in the statistical analysis plan. All diagnoses captured during a continuous inpatient spell will be considered, ensuring that relevant events are captured even if not the primary reason for hospital admission.

Prior to the primary analysis, survival status will be confirmed by cross-referencing UKRR and ONS data. For participants where no death has been recorded by either ONS or the UKRR at the end of the trial, follow-up time will be censored at the point of last known follow-up. Last known follow-up will be defined as whichever is most recent of either the date of last recorded UKRR-submitted dialysis results or last 6-monthly questionnaire follow-up. Data will be archived for a period of 5 years following the end of trial sponsor declaration.

### Confidentiality {27}

Study participants will provide explicit consent to the use of identifiable data for the purposes of the conduct of the study. Personal identifiable data (PID) will be stored separately from anonymised study data on a secure server hosted within the University of Cambridge School of Clinical Medicine Secure Research Computing Platform. PID will be accessible to the SIMPLIFIED trial team within the Cambridge Clinical Trials Unit, monitors, auditors and inspectors as required. It is necessary to (1) perform validation of NHS/CHI numbers and linkage to routinely collected datasets (NHS England, SAIL, eDRIS) and (2) to generate datasets with participant details for mail merge creation of questionnaires and is therefore imperative to the conduct of the study.

### Plans for collection, laboratory evaluation and storage of biological specimens for genetic or molecular analysis in this trial/future use {33}

Laboratory data will be obtained from the results of routinely collected blood tests via the UKRR on an on-going basis. Calcium and phosphate concentrations are measured regularly in all UK dialysis patients as part of standard care, and these data are already submitted to the UKRR. These results will be linked daily from the UKRR to the SIMPLIFIED secure data hosting area on all participants in the trial. Monitoring for hypercalcaemia will be driven by routine clinical practice and management based on local hypercalcaemia guidelines and practices.

## Statistical methods

### Statistical methods for primary and secondary outcomes {20a}

#### Primary outcome analysis

The primary efficacy analysis will be by intention-to-treat comparing patients randomised to cholecalciferol and those randomised to receive standard of care, adjusted for baseline characteristics including age, gender, diabetic status and length of time on dialysis. SIMPLIFIED is an event-driven trial and will continue until 2200 events (deaths) have occurred. Prior to the primary analysis, survival status will be confirmed by cross-referencing UKRR and ONS data. For participants where no death has been recorded by either ONS or the UKRR at the end of the trial, follow-up time will be censored at the point of last known follow-up. Last known follow-up will be defined as whichever is most recent of either the date of last recorded UKRR-submitted dialysis results or last 6-monthly questionnaire follow-up. The primary analysis will be carried out after the occurrence of 2200 events, using Cox proportional hazards regression modelling to test the effects of randomisation to cholecalciferol on all-cause mortality. The survival times will be summarised using Kaplan–Meier curves. All estimates of hazard ratios associated with treatment and baseline covariates will be provided with 95% confidence intervals and *p* values.

#### Secondary outcome analyses

Secondary assessments will evaluate the effect of cholecalciferol on quality of life using EQ-5D-5L data. Intention-to-treat analyses on the effect of randomisation to cholecalciferol on infections requiring admission, incidence of malignancy, fracture requiring admission and hospitalisation with cardiovascular events including atherosclerotic events, arrhythmias, heart failure and stroke will be carried out. Analysis of the time to first non-atherosclerotic cardiovascular event will be assessed.

Relevant secondary endpoints will be assessed using time to event analysis (Cox proportional hazards regression modelling). Time to event data will be summarised using Kaplan–Meier curves. Events that may occur multiple times (acute coronary syndrome, fracture) will be analysed using repeated events analysis. The treatment effect for secondary endpoints will be reported using point estimates, with nominal 95% confidence intervals and *p* values.

Cost-effectiveness will be gauged using a cost-effectiveness analysis (CEA) and cost-utility analysis (CUA). For both, the costing perspective will be that of the NHS (specifically that of secondary care—restricted to this perspective as resource data is only collected from Hospital Episode Statistics (HES) and related sources). In the CEA, outcomes will be compared in terms of life years gained (related to the primary outcome). For the CUA, outcomes will be compared using quality adjusted life years, calculated from EQ-5D-5L. For both analyses, a range of time frames (time horizons) will be considered, starting from a year and then at subsequent years for the range available within the data.

The overall conclusions of the study in terms of formal inference to reject or retain the null hypotheses associated with each endpoint will explicitly account for multiple testing to restrict the family-wise error rate (the overall chance of one or more type I errors) to below 5%.

### Interim analyses {21b}

A feasibility assessment will be carried out between months 12 and 15 of the trial. Feasibility will be predicated on recruitment rate (target 887 patients recruited after 12 months), and separation between arms by plasma vitamin D concentration after 4 months of treatment of 20 nmol/l.

### Methods for additional analyses (e.g. subgroup analyses) {20b}

Details will be documented in the statistical analysis plan which will be published as an appendix in the main results manuscript.

### Methods in analysis to handle protocol non-adherence and any statistical methods to handle missing data {20c}

The primary efficacy analysis will be conducted on the intention-to-treat (ITT) population, defined as all randomised participants, analysed according to their original treatment allocation, regardless of treatment adherence or post-randomisation events. For the primary endpoint (all-cause mortality), missing data are expected to be rare, as vital status will be ascertained for virtually all participants via routine data linkage and follow-up.

Further details in handling missing data are included in the statistical analysis plan which will be included as an appendix in the publication of the results manuscript. Further detail on the CEA and CUA will be detailed within the Health Economic Analysis Plan (HEAP).

### Plans to give access to the full protocol, participant-level data and statistical code {31c}

The full protocol is available on the study website and is submitted as supplementary material with this manuscript. De-identified participant data and statistical code will be shared on reasonable request to the corresponding author.

## Oversight and monitoring

### Composition of the coordinating centre and trial steering committee {5d}

#### Trial Management Group (TMG)

The TMG will meet at least weekly during initial set up, and then regularly as required to oversee the running of the trial. TMG members will include chief investigator, co-investigators, trial statistician(s), trial coordinator(s) and data manager(s) at the Cambridge Clinical Trials Unit (CCTU).

#### Trial Steering Committee (TSC)

The TSC is responsible for the review of the trial and related activities at regular intervals. The TSC also provides overall supervision for the trial, to ensure that it is conducted in accordance with the protocol and GCP and to provide advice through its independent chairman. The committee will aim to convene at regular intervals to review the data and discuss the recommendations from the IDMC. The details of the TSC are set out in the SIMPLIFIED Trial Steering Committee Charter, the composition of which will be approved by the NIHR.

### Composition of the data monitoring committee, its role and reporting structure {21a}

The Independent Data Monitoring Committee (IDMC) comprises an unblinded independent group, as defined in the SIMPLIFIED Data Monitoring Committee Charter document, which will define the role of the IDMC. The IDMC will have access to unblinded treatment allocation data, which it will use to review accumulating safety and efficacy data. The IDMC will meet regularly throughout the trial—from trial opening to final follow-up—and will make recommendations to the Trial Steering Committee regarding continuation, modification or termination of the trial.

### Adverse event reporting and harms {22}

This trial is categorised by the Medicines and Healthcare products Regulatory Agency as a type A study (no higher than the risk of standard medical care). Patients are followed up regularly at their local dialysis units throughout this study, and an ongoing evaluation of risk will continue throughout the recruitment period.

Serious adverse events (SAEs) will not be recorded and reported within the usual 24-h time period but will instead be captured using the routine data sources. SAE line listings will be periodically generated and reviewed by the chief investigator and reported to the sponsor. Serious adverse reactions or suspected unexpected reactions that are directly attributable to cholecalciferol will require reporting by site teams to the SIMPLIFIED trial office within 24 h of awareness.

Dialysis patients are intensively monitored as part of standard care with comprehensive biochemical and haematological tests being performed at least monthly. All dialysis data for each patient are reviewed by renal unit staff, typically in “sit down ward round” meetings, where clinical parameters and test results are reviewed and treatments adjusted. This approach will allow rapid detection of elevated calcium levels which is a potential concern with the administration of high-dose cholecalciferol, and clear guidance is provided to sites should hypercalcaemia occur via the hypercalcaemia (Fig. 1) and vitamin D toxicity (Fig. 2) algorithms.

### Frequency and plans for auditing trial conduct {23}

The sponsor will complete risk-based and targeted onsite and remote monitoring of trial sites based on the trial risk assessment, patient safety and regulatory compliance. No formal regulatory or registry audits are planned, but should a monitoring visit or audit be requested, the trial team will make the required trial documentation and source data available to the auditor’s representative.

### Plans for communicating important protocol amendments to relevant parties (e.g. trial participants, ethical committees) {25}

Protocol amendments must be reviewed and agreement received from the sponsor for all proposed amendments prior to submission to the Research Ethics Committee (REC) and/or Medicines Health and Regulatory Authority (MHRA). The only circumstance in which an amendment may be initiated prior to REC and/or MHRA approval is where the change is necessary to eliminate apparent, immediate risks to the participants (Urgent Safety Measures). In this case, accrual of new participants will be halted until the REC and/or MHRA approval has been obtained. All correspondence with the REC will be retained in the Trial Master File/Investigator Site File. All REC/HRA/MHRA approvals will be shared with trial sites for local C&C approvals (as required) prior to trial site implementation. A complete list of all protocol amendments will be included in the final clinical study report.

### Dissemination plans {31a}

On completion of the trial, the data will be analysed and tabulated and a Final Study Report prepared. The results of this trial may be presented at scientific meetings and will subsequently be published in peer review journals. The NIHR Health Technology Assessment (HTA) requires notification of all outputs arising from the trial 28 days before publication. Results will be shared with participants in an accessible format.

## Discussion

Dialysis is a life-sustaining treatment, but there is a major unmet need to improve survival; one third of patients have died within 3 years of commencing renal replacement therapy, an outcome that is worse than most common cancers. Many interventions are employed in dialysis, aiming to improve patients’ survival and quality of life, but with little supporting evidence. Vitamin D deficiency is observed in the majority of patients on dialysis, and there is strong epidemiological evidence that low levels of vitamin D are associated with increased risk of all-cause mortality, cancer, cardiovascular events and infection. However, the impact of population-wide vitamin D replacement on dialysis outcomes is unknown.

Building on the lessons learned from the design and conduct of earlier trials using routinely collected electronic health care data to capture outcomes, the SIMPLIFIED trial has a pragmatic study design to address the key question of whether high-dose cholecalciferol improves survival in patients on dialysis [21]. The study was designed to place minimal burden on patients and dialysis unit staff and to utilise routinely collected data and centralised data collection to report endpoints wherever possible. Consequently, there were no specific study visits after the initial baseline visit, which enabled the trial to continue throughout the COVID-19 pandemic when most studies had to pause due to diversion of resources.

Patients receiving dialysis are intensively supervised as part of routine care and have frequent routine blood monitoring which automatically flows to the UKRR primarily for audit purposes. This established infrastructure means that employing methods to harness routinely collected data in clinical trials is ideally suited to this patient population. By harnessing the efficiencies of using data from such sources, with the available budget, it is feasible to enrol a large number of patients receiving dialysis into this study and follow for several years to definitively answer the question of whether high-dose cholecalciferol improves survival for patient on dialysis. Cholecalciferol is a low-cost and easy to administer intervention, and should it show benefit, then these findings should be readily translatable to dialysis patients more broadly both in the UK and beyond.

It is anticipated that the SIMPLIFIED trial will pave the way for future registry trials in the UK as it will serve as a vehicle to develop the infrastructure and experience needed to support more complex trials using this approach. In addition, since all relevant data will continue to be routinely captured on all participants beyond the end of the follow-up period, it offers a very cost-effective approach for extended follow-up and evaluation of long-term outcomes of participants.

## Trial status

Recruitment to the SIMPLIFIED trial commenced in March 2017, but has experienced significant delays due to the COVID-19 pandemic. The study is now active in 55 UK renal units. The current protocol is version 11.0 dated 6th February 2025. As of 1 st May 2025, 3800 patients have been recruited. Recruitment is expected to close in Q3 2025 and follow-up be completed by the end of Q4 2025. Analysis is planned for Q2 2026.

## Data Availability

When the database from the trial is locked, analysed and published, we will endeavour to make data available upon reasonable request and pursuant to contractual agreements*.*

## Abbreviations

ACS: Acute coronary syndrome
C&C: Capacity and capability
CCTU: Cambridge Clinical Trials Unit
CEA: Cost-effectiveness analysis
CUA: Cost-utility analysis
CHI: Community Health Index
CKD: Chronic kidney disease
eCRF: Electronic case report form
eDRIS: Electronic Data Research and Innovation Service
Eq5D: Euro-Qol 5 Dimension
ESKD: End stage kidney disease
HES: Hospital Episode Statistics
HRQoL: Health-related quality of life
HTA: Health Technology Assessment
ISRCTN: International Standard Randomised Controlled Trial Number
KDIGO: Kidney Disease Improving Global Outcomes
MHRA: Medicines Health and Regulatory Authority
NHS: National Health Service
NIHR: National Institute of Health Research
ONS: Office of National Statistics
PI: Principal investigator
PID: Personal identifiable data
REC: Research Ethics Committee
SAE: Serious adverse event
SAIL: Secure Anonymised Information Linkage
SAR: Serious adverse reaction
SUSAR: Suspected unexpected serious adverse event
UK: United Kingdom
UKRR: UK Renal Registry
